# An investigation of the relationship between female university students' breast cancer risk factors and their health beliefs about breast self-examination

**DOI:** 10.4314/ahs.v23i4.28

**Published:** 2023-12

**Authors:** Zehra Golbasi, Birnur Yeşildağ, Nermin Altunbaş

**Keywords:** Breast cancer, breast cancer awareness, risk factors, health belief

## Abstract

**Purpose:**

The purpose of this study is to determine the relationship between female university students' breast cancer risk factors and their health beliefs about breast self-examination (BSE).

**Methods:**

The sample of this descriptive and correlational study was 389 female students who were determined by stratified sampling method. Data were collected through the Socio-demographic Characteristics and Breast Cancer Risk Factors Determination Form and the Champion's Health Belief Model Scale (CHBMS). Data obtained from the study were analysed using SPSS for Windows 16.0 program. Data analysis was performed using percentage distributions, z test, and Spearman correlation analysis. Statistical significance was accepted p<0.05.

**Results:**

While a positive correlation was detected between students' perceived breast cancer risk for themselves and perceived susceptibility, severity, benefit, self-efficacy related to BSE and health motivation mean scores, a negative and significant correlation was detected with perceived barriers to BSE mean score (p<0.05).

**Conclusion:**

Results showed that although female university students had some risk factors in terms of breast cancer, they have low levels of perceived risk factors for themselves.

## Introduction

Breast cancer is ranked the first among all cancers, and the incidence rate standardized by both genders and each age group is reported to be 47.8 in one hundred thousand and mortality rate is 13.6 in one hundred thousand.^1^-^2^ The breast cancer standardized incidence rate was 47.7% in Turkey according to both genders and each age group.^1^ There are many modifiable and non-modifiable factors that increase the risk of developing breast cancer.^3^ Factors such as age, gender, genetics and family history, atypical hyperplasia, race, dense breast tissue, benign breast diseases, having received radiation in the breast area before, early menarche are classified as unchangeable risk factors.^4^-^5^-^6^ While changeable factors include factors such as postmenopausal hormone treatment,^4^ breastfeeding,^4^-^7^ alcohol,^4^-^5^-^8^ nutrition and obesity,^4^ physical activity,^7^ Type II diabetes,^10^ uncertain risk factors are classified as nutrition,^12^-^13^ chemicals in the environment, and smoking.^13^ The best way of fighting cancer, including breast cancers with rapidly increasing rates, is to control the known risk factors.^3^-^13^ In this respect, the American Cancer Society emphasizes the importance of primary and secondary prevention methods for breast cancer.^14^ While the primary protection methods include taking measures for breast cancer risk factors and preventing the development of breast cancer,5 secondary protection methods include decreasing morbidity and mortality rates with early diagnosis, screenings, and effective treatment.^14^

Screening programs used for the early diagnosis of breast cancer mainly include BSE, Clinical Breast Examination, and Mammography.^5^-^12^ Breast self-examination is defined as women's checking their breasts and surrounding areas at certain intervals regularly to determine changes in the shape or the presence of an unusual lump to detect breast cancer in the early period.^12^ The importance of BSE increases when it is considered that the first symptom of breast cancer is a lump felt by hand, and around 80% of women noticed a lump in their breast firstly by themselves. According to the Turkish national breast cancer screening standards, women are recommended to have BSE regularly starting from the age of 20.^1^ However, the literature reports that women have low levels of demonstating early diagnosis behaviours against breast cancer.^15^-^16^-^17^ According to the Annual Health Statistics 2020 data, 22,1% of women aged 15 and over in Turkey performed BSE every month regularly and 4,3% had never performed BSE.^1^ Some of the reasons for low BSE rates include neglect, lack of knowledge, and the fear of finding a lump in the breast and having breast cancer.^17^-^18^ On the other hand, women's health beliefs are another factor that affects their behaviours for early diagnosis.^17^-^19^ The Health Belief Model (HBM) is commonly used in explaining breast cancer screening behaviours. HBM is used in understanding the factors that motivate individuals for protective behaviours and how to act for screening for early diagnosis.^20^ According to the model, the probability of performing BSE increases as the woman perceives the breast cancer threat closer.^21^

In our country, where breast cancer is ranked the first among women's causes of diseases and death, there is a need for increasing awareness of women about breast health and breast cancer starting from young ages.^22^ Although female university students have relatively lower risks in terms of breast cancer, they can be considered the target population in terms of determining the risk factors in the early period, informing them about BSE and other screening programs, and raising awareness about breast cancer. This study aims to determine the relationship between female university students' breast cancer risk factors and their health beliefs about breast self-exam (BSE).

## Methods

### Study design

This study utilized a descriptive and correlational study design.

### Target population and the sample

The population of the research consisted of 3710 senior female students studying in the daytime education of undergraduate programs in the central campus of a university in Turkey. Of all these students, 544 were enrolled in health-related fields, 797 were enrolled in science fields, 576 were enrolled in the education field, and 1793 were enrolled in the social sciences field. The sample size was calculated using the formula utilized for determining the prevalence of cases when the number of the target population is known and was found as 389 (N=3710, t=1.96, d=0.05, p=0.50, q=0.50). The research was completed with 389 female students studying in the last year of Sivas Cumhuriyet University undergraduate programs. Fields in which female students were enrolled were seen as a stratum, and the number of students to be included from each field was calculated by the strata weight (Field of Health Sciences:57, Field of Science:84, Field of Education Sciences: 61, and Field of Social Sciences:187). The entire number of participants required to be included in the sample group has been reached.

### Data collection

Data were collected through the “Socio-demographic Characteristics and Breast Cancer Risk Determination Form” and “Champion's Health Belief Model Scale”. Socio-demographic characteristics and breast cancer risk determination form

The first part included 9 questions that aimed to determine female students' characteristics such as age, department, family type, economic condition, etc. The second part included 19 questions that aimed to determine the presence of some variables considered to be risk factors in terms of breast cancer (age of menarche, weight etc.). The last question of the form was a numeric scale ranging from 1 to 10 for determining the breast cancer risk level (1= lowest- 10= highest).^23^-^24^-^25^-^26^

### Champion's health belief model scale (CHBMS)

The scale was developed by Victoria Champion in 1984 to measure beliefs about breast cancer and BSE and includes five sub-scales and 39 items.^27^ Champion (1993) revised the scale and included the self-efficacy/confidence sub-scale.^28^ For the subscales of CHBMS the internal reliability Cronbach alpha coefficient was ranged from 0.69 to 0.90 and test-retest reliability range from 0.45 to 0.70 Reliability and validity of the scale were performed by Karayurt (2003) and Gözüm and Aydin (2003). This study utilized the scale adapted by Karayurt, which included six sub-scales and 42 items.^29^ The items are responded on a 5-point Likert scale. Each sub-scale is scored separately and is not combined into one total score. Cronbach alpha reliability rates of subscales of CHBMS have been found 0.58-0.85 at adaptation of Karayurt and the reliability of test-re-test has been found 0.89-0.99. In this study; the Cronbach's alpha values ranged between 0.73 and 0.96.

### Data collection

Female students were given information about the purpose of the study at a time and place indicated by the department administration, and written consent was received from those who indicated their volunteer participation. There was no incentive to participate in the study. Data collection forms were distributed to students between February 2018 and April 2018, and they were provided to fill in individually after necessary explanations were made. All participants filled out the forms completely.

### Statistical analysis

Data obtained from the study were analysed in SPSS for Windows 16.0 program. Data analysis included percentage distributions, z test, and Pearson correlation analysis. Statistical significance was accepted p<0.05.

## Results

The average age of the participating students was 22.5, and 97.2% were single, 95.1% did not work, and 78.8% defined their economic condition as medium-level. Besides, 80.7% of the students reported to have received information about breast cancer, and the sources of information included primarily health personnel (46.3%), internet (38.6%), and written/visual media (Table 1).

**Table 1:** Distribution of the Students by their Descriptive Characteristics (n=389)

Variables	Number	%
**Marital status**		
Single	378	97.2
Married	11	2.8
**Working or not**		
Working	19	4.9
Not working	370	95.1
**Economic condition**		
High	68	17.5
Medium	306	78.7
Low	15	3.9
**Having received information about breast cancer**		
Yes	314	80.7
No	75	19.3
**Sources of information about breast cancer** *		
Health personnel	180	46.3
Internet	150	38.6
Written and visual media	111	28.5
Family, relatives	46	11.8
Friends	37	9.5
Average age: 22,5 (SD= 2.45)		

* *There is more than one response; percentages are based on “n”*

When the factors that could be risk for breast cancer were analysed, it was found that 10% of the participants had breast cancer history among their first-degree relatives (mother, grandmother, aunt, sister/elder sister); 4.1% had a menarche age of 11 and below; 2.1% had had breast biopsy before; and 12.9% were overweight according to Body Mass Index (BMI). Besides, 26.7% of the students smoked, 82.8% did not exercise regularly, 49.6% used underarm anti-sweat lotion every day, 34.7% consumed red meat three times and more a week, and 47.8% consumed margarine/animal fat three times and more a week. Students were asked to mark their perceived breast cancer risk level for themselves on a scale between 0 and 10, and the perceived breast cancer risk mean score was found 2.63 (SD=2.14) (Table 2).

**Table 2:** Distribution of Students by Factors that could be risk for Breast Cancer (n=389)

Variables	Number	%
**Family history of breast cancer**		
Yes	40	10
No	349	90
**Presence of a family member with breast cancer (n=40)**		
Mother	2	5
Sister	3	7.5
Aunt	17	42.5
Grandmother	18	45
**Age of menarche**		
11 and below	16	4.1
12-15	345	88.7
16 and above	28	7.2
**Having had breast biopsy**		
Yes	8	2.1
No	381	97.9
**Body Mass Index (BMI)**		
Obese	48	12.3
Overweight	50	12.9
Normal weight	196	50.4
Thin	95	24.4
**Smoking**		
Smoking	104	26.7
Not smoking	285	73.3
**Exercising regularly**		
Yes	67	17.2
No	322	82.8
**Using underarm anti-sweat lotion**		
Yes	193	49.6
Sometimes	142	36.5
No	54	13.9
**Consuming red meat**		
Once a week	224	57.6
Three times and more a week	135	34.7
Never	30	7.7
**Consuming Margarine/Animal Fat**		
Once a week	154	39.6
Three times and more a week	186	47.8
Never	49	12.6
**Perceived breast cancer risk mean score: 2.63 (SD=2.14)**		

*SD: Standard deviation*

An analysis of s tudents' CHBMS mean scores indicated that perceived susceptibility related to breast cancer mean score was 7.22±2.49; perceived severity related to breast cancer mean score was 22.32±6.17, perceived benefits of Breast Self-Exam (BSE) mean score was 15.32±3.79, perceived barriers to BSE mean score was 25.48±7.45, perceived self-efficacy towards performing BSE mean score was 34.17+8.84, and the health motivation sub-scale mean score was 26.21+5.49 (Table 3).

**Table 3:** Champion's Health Belief Model Scale Sub-Scale Mean Scores

Champion's Health Belief Model Sub-scales (CHBMS)	Number of Items	Min-Max values to be obtained from the scale	X ±SD
Perceived Susceptibility related to Breast Cancer	3	3-15	7.22±2.49
Perceived Severity related to Breast Cancer	7	7-35	22.32±6.17
Perceived Benefits of BSE	4	4-20	15.32±3.79
Perceived Barriers to BSE	11	11-55	25.48±7.45
Perceived Self-efficacy towards performing BSE	10	11-50	34.17±8.84
Health Motivation	7	7-35	26.21±5.49

*Min: Minimum, Max: Maximum, SD: Standard deviation*

CHBM perceived susceptibility related to breast cancer sub-scale mean score was higher in students who had a family history of breast cancer in comparison to those who did not (p<0.05), and the health motivation sub-scale mean score was found to be low (p<0.05). CHBM perceived benefits of performing BSE, self-efficacy towards performing BSE, and health motivation sub-scale mean scores were higher (p<0.05), and perceived barriers to BSE mean score was lower (p<0.05) in students who reportedly received information about breast cancer before in comparison to those who did not. Students who received information about breast cancer from health personnel had higher mean scores in the CHBM Perceived Benefits of performing BSE; Perceived Barriers to performing BSE mean score was higher in students who did not receive information from health personnel (p<0.05). There was no statistical difference between the students' age of menarche, having had breast biopsy, body mass index, smoking, exercising regularly, using underarm anti-sweat lotion, consuming red meat and consuming margarine/animal fat the mean scores of the CHBM sub-dimension (Table 4).

**Table 4:** Students' CHBMS sub-scale scores according to their characteristics

Variables	Perceived Susceptibility related to breast cancer	Perceived Severity related to breast cancer	Perceived Benefits of perfor ming BSE	Perceived Barriers to performing BSE	Perceived self-efficacy towards performing BSE	Health Motivation
	X ±SD	X ±SD	X ±SD	X ±SD	X ±SD	X ±SD
**Family history of breast cancer**						
Yes No	8.69±2.26 7.06±2.47	23.69±6.21 22.16±6.15	15.39±4.05 15.32±3.77	27.17±7.04 25.29±7.48	34.53±7.77 34.13±8.96	23.79±5.80 26.48±5.40
Statistical Test	z=-3.731 p=0.000	z=-1.061 p=0.289	z=-,422 p=0.673	z=-1.723 p=0.085	z=-,173 p=0.863	z=-2.828 p=0.005
**Receiving information about breast cancer**						
Yes No	7.33±2.46 6.80±2.58	22.17±6.14 22.94±6.27	15.67±3.64 13.85±4.07	25.10±7.64 27.06±6.40	35.58±8.40 28.25±8.22	26.64±5.46 24.41±5.30
Statistical Test	z=-1.544 p=0.123	z=-,961 p=0.337	z=-3.685 p=0.000	z=-2.696 p=0.007	z=-6.702 p=0.000	z=-3.227 p=0.001
**Receiving information from health personnel**						
Yes No	7.36±2.37 7.28±2.59	22.47±5.56 21.76±6.85	16.13±3.55 15.05±3.68	23.60±7.22 27.13±7.74	36.20±8.20 34.76±8.63	26.76±5.49 26.49±5.43
Statistical Test	z=-.036 p=0.972	z=-1.081 p=0.280	z=-4.198 p=0.004	z=-4.198 p=0.000	z=-1.697 p=0.090	z=-.433 p=0.665

*SD: Standard deviation; Z: Z-test in independent groups*

A statistically significant and positive relationship was detected between students' perceived breast cancer risk mean score and perceived susceptibility, perceived severity, and perceived benefits of and perceived self-efficacy towards performing breast self-examination (p<0.05) (Table 5).

**Table 5:** Relationship between students' perceived breast cancer risk mean score and champion's health belief model mean scores

	Champion's Health Belief Model Sub-scales (CHBMS)					
	Perceived susceptibility related to breast cancer	Perceived severity related to breast cancer	Perceived benefits of performing BSE	Perceived barriers to performing BSE	Perceived self-efficacy towards performing BSE	Health motivation
**Perceived breast cancer risk score**	r=0.545 p=0.000	r=0.355 p=0.000	r=0.142 p=0.005	r=-0.028 p=0.581	r=0.119 p=0.018	r=0.015 p=0.775

*r: Spearman's correlation analysis*

## Discussion

This study, which aimed to determine female university students' risk factors, found that the majority of the participants received information about breast cancer; their sources of information about breast cancer were visual and written media and friends/relatives, yet the main source of information was health personnel. Some studies conducted with different populations reported less information received by women about breast cancer,^30^-^31^ while some others reported more information about breast cancer and early diagnosis practices.^32^-^33^ Several studies indicated that the sources of information about breast cancer were television, newspaper/journals, friends/relatives, and health personnel was reported less than other sources.^15^-^34^-^35^ Similar to the present study, Sadıç (2019) reported that the source of information about breast cancer and early diagnosis practices were health personnel for 40.8% and television, books, and journals for 11,2%.^32^ In this study, participating students' primary source of information was health personnel, which is a positive finding. Obtaining information from reliable sources is important in terms of the accuracy of the information. This finding can be related to the high education level of participating students.

Perceived breast cancer risk for themselves was found to be low in the majority of participating students. Similar to the women's perceived breast cancer risk for themselves was reported to be low in other studies.^36^-^37^ Perception of low risk in terms of breast cancer can be considered to be a negative factor to affect early diagnosis and screening behaviours. Women whose first-degree relatives have breast cancer are at higher risk in terms of breast cancer.^11^-^12^ While this risk increases approximately two times if one of the first-degree relatives has breast cancer, it increases around three times if two of the first-degree relatives have breast cancer.^12^ In this study, 10% of students (n=40) had a family history of breast cancer, and breast cancer was seen mostly in grandmothers (45%) and aunts (42.5%). Demir Yıldırım and Özaydin 35 found that 6,9% of women had breast cancer history in their first-degree relatives, and these relatives were mothers for 52,5%, sisters for 41,8% and daughters for 6,7%.

Hormonal condition is another effective factor that plays a role in the development of breast cancer. Generally, having menarche one year later decreases the breast cancer risk by 20%.^12^ Having menarche before the age of 12 increases the breast cancer risk.^36^ In their study that included 210 patients and aimed to investigate factors considered to be risk factors for breast cancer, Aydogan et al. detected no significant differences between patients' age of menarche and the menarche age of the participants in the control group.^24^ This study found that 4.1% of students had menarche at the age of 11 and below. This result can be considered to be a warning sign for including students in protection programs against breast cancer.

There is a limited number of studies that prove the relationship of breast cancer with smoking, a changeable risk factor. A study reported that starting smoking before the first pregnancy and smoking for a long time increased the risk of breast cancer slightly.^12^ In this study, one-fourth of participating students were found to smoke. Other similar studies reported low levels of smoking.^39^-^40^ Increased breast cancer risk in obese women is reported to be related to estrogen released from large fatty tissues.^12^ A positive relationship is reported between breast cancer risk and body mass index of ≥30kg/m^2^.^41^ Of all the participating students, 12.9% were overweight according to Body Mass Index (BMI). The majority of the students had normal BMI, which can be associated with high education level and more importance given to body image in this age group.

Exercising regularly, an important way of preventing obesity, is reported to decrease women's breast cancer risk by 10-20%,^42^-^43^ and 150 minutes of moderate level physical activity is recommended weekly to decrease the risk of breast cancer.^9^ The majority of the students in this study (82.8%) reported that they did not exercise regularly. Similarly, Arslanoğlu et al. (2021), 24.3% of 2923 university students determined that she did regular physical activity.^44^ As the study group is composed of young individuals, they can be considered an important group in terms of raising awareness about exercising. In the study, it was determined that 49.6% of the students used underarm antiperspirants. There is insufficient evidence in the literature to show that underarm antiperspirants cause breast cancer.^45^-^46^

A high level of a fatty diet is considered to increase breast cancer.^5^-^11^-^26^ A diet with high fat leads to obesity and increases the level of insulin released. Besides, fatty tissue holds estrogen, which causes more endogen estrogen release. Besides, the fatty tissue releases estrogen independently.^4^-^9^ In this study, 34.7% of participating students were found to consume red meat three times and more a week, and 47.8% consumed margarin/animal fat three times and more a week. Anderson et al. reported no association between red meat consumption and breast cancer risk in a meta-analysis of 11 prospective cohorts, whereas Lo et al. (2020) found that red meat consumption increased the risk of invasive breast cancer.^47^-^48^

An analysis of university students' CHBM mean scores and standard deviations indicated that the highest score was received from the perceived self-efficacy/confidence scale (34.17±8.84), and the lowest score was received from the perceived susceptibility sub-scale (7.22±2.49). Perceived benefits of BSE mean score (15.32±3.79) was found to be lower than the perceived barriers to BSE mean score (25.48±7.45). Perceived barriers are reported to be the most important factor for realizing a behaviour; perceived barriers can change and perceived benefits can increase with education, consultancy, notices, and interventions that enhance access to health.^20^ Kulakçı et al. (2015), in the nursing students in their study, it was determined that students; seriousness perception was moderate, health motivation, BSE benefits and BSE self-efficacy perceptions were high, and BSE barriers and sensitivity perceptions were low.^49^ Kulakçı Altıntaş and Korkmaz Aslan (2019), it was found seriousness, health motivation, breast self examination benefits and breast self examination self-efficacy perceptions of the midwives and nurses were moderate, and susceptibility, breast self examination barriers and breast fatalism perceptions were low.^50^ High levels of perceived barriers in this study indicate low levels of breast cancer awareness among university students.

Students who have a family history of breast cancer had higher CHMS perceived susceptibility related to breast cancer sub-scale mean score (p<0.05) and lower health motivation sub-scale mean scores (p<0.05) than those who did not have a family history of breast cancer. Studies reported higher perceived susceptibility of women who had a family history of breast cancer.^49^-^51^ Susceptibility contains beliefs about the probability of having breast cancer. High perceived susceptibility of students indicates performing screening behaviours better due to the higher perception of breast cancer as a risk. Students who reported to receive information about breast cancer before were found to have higher CHBM perceived benefits of BSE, self-efficacy towards performing BSE, and health motivation sub-scale mean score (p<0.05) and lower perceived barriers to BSE mean score than students who did not receive information (p<0.05). Erbil and Bolukbas reported higher perceived benefits of BSE, perceived self-efficacy towards BSE, and health motivation of those who have information about breast cancer and lower perceived barriers to BSE.^52^ Duman et al. found that women who had information about breast cancer had higher caring and perceived self-efficacy towards BSE.^53^ Lower perceived barriers of students are highly important in terms of increasing BSE ratios and thus perceived benefits of BSE and perceived self-efficacy towards BSE. This study found that students who received information about BSE from health personnel had higher CHBM perceived benefits of BSE, and perceived barriers to BSE mean score was high in those who did not receive information from health personnel (p<0.05). Studies on the early diagnosis of breast cancer should consider students' health beliefs and benefit from receiving information from health personnel to decrease perceived barriers.

## Conclusion and recommendations

Results obtained from this descriptive and correlational study show that female university students have unchangeable risk factors such as a family history of breast cancer and age of menarche as well as changeable lifestyle risk factors such as BMI, smoking, nutrition, exercise, and using underarm anti-sweat lotions. On the other hand, their perceived breast cancer risk factor for themselves was relatively low. Receiving information about breast cancer and having a family history of breast cancer affect students' health beliefs about breast cancer. Students' health belief perceptions should be taken into consideration in the demonstration of early diagnosis behaviours, and trainings should be given on this issue to affect health beliefs. These trainings should enhance the demonstration of behaviours by affecting perceived susceptibility, severity, benefits, and barriers as components of the health belief model, as well as health motivation and self-efficacy beliefs.

## Limitations of the study

The most important limitation in the study is that it is a single-center study. Because the study has a small sample size and reflects only the results of this region, the results obtained from this study carried out at the are applicable only to the participating female university students.
